# A Single-Center Study on Clinico-Radiological Evaluation of Inguino-Scrotal or Labial Swellings Presenting to a Tertiary Care Set-Up in Central India

**DOI:** 10.7759/cureus.58162

**Published:** 2024-04-13

**Authors:** P Vinoth, Roshan Chanchalani, Radha Sarawagi, Meena Kumari, Bharati Pandya

**Affiliations:** 1 Department of General Surgery, All India Institute of Medical Sciences, Bhopal, Bhopal, IND; 2 Department of Paediatric Surgery, All India Institute of Medical Sciences, Bhopal, Bhopal, IND; 3 Department of Radiodiagnosis and Imaging, All India Institute of Medical Sciences, Bhopal, Bhopal, IND

**Keywords:** radiology, swelling, groin, inguino-labial, inguino-scrotal

## Abstract

Introduction

The inguino-scrotal/labial region, anatomically defined as the juncture where the thigh meets the lower abdomen, encompassing the ipsilateral scrotal area in men and the inguino-labial area in women, exhibits a broad spectrum of masses. Traditionally, these swellings were clinically diagnosed with high accuracy, primarily due to the prevalence of simple hernias and hydroceles. However, contemporary observations reveal a surge in complex inguino-scrotal/labial swellings, particularly in referral hospitals, necessitating additional radiological and imaging modalities for precise diagnosis. Our interest in this subject was sparked by the escalating numbers of intricate inguino-scrotal/labial masses encountered in our medical setting, posing challenges for clinical diagnosis in both pediatric and adult populations.

Materials and methods

A prospective, observational study was conducted over two years (August 2021 to March 2023) involving 210 patients presenting with inguino-scrotal/labial swellings at our institute. Clinical data were meticulously collected using a designed pro forma, following informed consent procedures.

Results

Among the 210 patients with inguino-scrotal/labial swellings, males predominated (194) compared to females (16). The paediatric age group comprised 84 patients, while 126 were adults. Radiological investigations played a crucial role in diagnosing 40 patients and provided significant additional information in 12 cases. Radiological investigations contributed to the diagnosis in 52 patients (24.76%). The study revealed a spectrum of new entities in the inguino-scrotal/labial region, including malignancies, lymph nodal masses in the groin, and vascular, inflammatory, and congenital lesions, which might have been overlooked if solely relying on clinical parameters for diagnosis.

Conclusion

Inguino-scrotal/labial swelling patients, especially those facing diagnostic dilemmas or harbouring complex lesions, should undergo radiological assessment as an indispensable criterion, particularly when such facilities are readily accessible.

## Introduction

A diverse array of masses can manifest in the inguino-scrotal/labial region, defined anatomically as the area that is the junction between the thigh and lower abdomen. This encompasses the scrotal region in men and the inguino-labial region in women on the same side [1]. Recently, there has been a notable increase in complex inguino-scrotal/labial swellings, particularly in referral hospitals. Many of these cases require additional radiological and imaging modalities for an accurate diagnosis [2]. The types of masses occurring in this region exhibit considerable diversity, with approximately 75% of them localized in the inguinal region alone [3]. Inguinal masses may arise both above and below the inguinal ligament, categorizable into five major groups: congenital abnormalities, non-congenital hernias, vascular conditions, infectious or inflammatory processes, and neoplasms. Congenital entities show a high prevalence of inguinoscrotal swellings [4]. Historically, these swellings have been clinically diagnosed with great accuracy, primarily consisting of simple hernias and hydroceles. However, the complexity of inguino-scrotal/labial swellings has increased in recent times, particularly in tertiary care settings where the presentation of both adult and paediatric cases poses a diagnostic challenge. This complexity arises from factors such as previously operated swellings, recurrences, emergency presentations of acute painful swellings, neoplastic aetiology, and various co-morbid conditions [5]. This shift has led to the essential role of radiological and imaging modalities, including ultrasonography, computed tomography (CT), and magnetic resonance imaging (MRI), in detection, diagnosis, staging, and surveillance [1, 2]. Medico-legally, an accurate diagnosis is crucial before deciding on treatment options.

Ultrasound (USG) aids in determining the echogenicity of masses, vascular status, and the presence of malignancy risk [2,3]. CT is valuable for assessing traumatic injuries and cancer staging, often combined with positron emission tomography (PET) and CT. MRI offers an excellent characterization of inguino-scrotal/labial focal lesions and a valuable depiction of the local extent of pathological processes, emerging as a key problem-solving imaging modality when sonography results are inconclusive [2]. Optimized treatment options and surgical techniques are tailored to the specific type of inguino-scrotal/labial mass. A comprehensive understanding of the anatomy of the inguino-scrotal/labial region, assessed through clinical, radiological, and even laparoscopic modalities, has become essential. The added benefits of radiological and laparoscopic modalities include determining the laterality of swellings, which can be challenging based solely on clinical parameters, such as hernias and lymph nodal status in perineal malignancies. A detailed history and physical examination, complemented by accurate imaging analysis, contribute to a thorough understanding of the clinical presentation. This approach facilitates the recognition and treatment of both common and uncommon masses in the inguino-scrotal/labial region. Our interest in this topic stems from the increased number of complicated, difficult-to-diagnose cases encountered in our setup in both paediatric and adult populations visiting our outpatient department (OPD). Additionally, we observed a scarcity of literature available for such an evaluation.

## Materials and methods

Study design and setting

The aforementioned research was executed and documented as an observational study, adhering to the principles delineated in Strengthening the Reporting of Observational Studies in Epidemiology (STROBE). Approval for the study was obtained from the Institutional Ethics Committee, referencing IHEC-PGR/2021/PG/Jan/20. The investigation transpired within the Department of General Surgery and Paediatric Surgery at our institute, following clearance from our Institutional Ethics Committee board. All patients meeting the inclusion criteria and presenting with inguino-scrotal swelling at the OPD and the emergency department were incorporated into the study. Before inclusion, each patient received an information sheet and an explanation of the study. Informed written consent was acquired from every patient before the commencement of the study. For minors, i.e., patients under 18, consent was obtained from their parents or guardians.

Study participants

The overall sample size was established at 195, taking into account an estimated prevalence (P) of 0.15, a 95% confidence level (Z = 1.96), and a desired precision of 0.05 (5%). Consequently, the calculated study population was determined to be 210. The inclusion criteria encompassed individuals of both genders, including paediatric and adult populations, and involved routine and emergency cases presenting with inguino-scrotal/labial swellings. Exclusion criteria comprised patients who did not consent to participate in the study.

Methodology

A comprehensive clinical examination was conducted on all patients following a detailed history-taking process. Routine haematological and biochemical investigations were performed. In cases where there was even minimal uncertainty based on clinical examination, imaging studies such as ultrasonography were conducted. The basic radiological modalities were ultrasonography and contrast-enhanced CT scans, which got us to the diagnosis in almost all the cases. Few require advanced radiological modalities like MRI. Contrast-based radiography was otherwise not used. Further imaging studies were pursued if the initial diagnosis using this modality proved inconclusive. Detailed records were kept regarding conservative, interventional, or operative management approaches. The management details and outcomes were meticulously documented. Patients were regularly monitored through follow-up visits in general surgery, pediatric surgery, the OPD, or via telecommunication. A thoughtfully designed proforma, which was designed by us after the thesis review committee meeting in which it was approved by our department, was utilized to record systematically and document communications related to the study. The study was conducted over a timeframe spanning from August 2021 to December 2023.

Statistical analysis

Data collection utilized a paper-based proforma that comprehensively captured patient details, medical history, clinical examination findings, and imaging results. Subsequently, the collected data underwent entry into an MS Excel spreadsheet (Microsoft® Corp., Redmond, WA). The analysis phase involved the utilization of SPSS software, specifically version 23 from SPSS Inc., based in Chicago, USA. For calculating the significance, an unpaired t-test was used. The level of significance was fixed at p<0.05 for statistical tests.

## Results

Paediatric and adult populations, as well as patients presenting with co-morbidities, are displayed in Table 1.

**Table 1:** Sex-wise distribution of patient characteristics. ^a^n (%). ^b^p-value was calculated using an unpaired t-test. N: number of patients; IQR: interquartile range; SD: standard deviation.

Characteristics	N = 210^a^	Female, N = 16^a^	Male, N = 194^a^	p-value^b^
Age (years)				
Median (IQR)	31.00 (6.00, 55.75)	10.50 (6.75, 23.25)	34.50 (6.00, 56.75)	0.054
Range	0.04, 97.00	0.04, 60.00	0.16, 97.00	
Mean (SD)	32.19 (25.17)	18.88 (19.25)	33.28 (25.33)	
Age groups				
<1 year	12 (5.71%)	1 (6.25%)	11 (5.67%)	0.3
>1–10	59 (28.10%)	7 (43.75%)	52 (26.80%)	
11–20	18 (8.57%)	4 (25.00%)	14 (7.22%)	
21–30	16 (7.62%)	0 (0.00%)	16 (8.25%)	
31–40	18 (8.57%)	1 (6.25%)	17 (8.76%)	
41–50	23 (10.95%)	1 (6.25%)	22 (11.34%)	
51–60	28 (13.33%)	2 (12.50%)	26 (13.40%)	
61–70	26 (12.38%)	0 (0.00%)	26 (13.40%)	
71–80	8 (3.81%)	0 (0.00%)	8 (4.12%)	
81–90	1 (0.48%)	0 (0.00%)	1 (0.52%)	
91–100	1 (0.48%)	0 (0.00%)	1 (0.52%)	
Patient category				
Paediatric	84 (40.00%)	10 (62.50%)	74 (38.14%)	0.056
Adult	126 (60.00%)	6 (37.50%)	120 (61.86%)	
Comorbidities				
Absent	170 (80.95%)	16 (9.4.00%)	156 (91.76%)	0.6
Present	40 (19.04)	0 (0.00%)	40 (100%)	

As a referral centre in Central India, our study also took into account the presence of co-morbidities. Among the participants, 40 individuals (19.04%) were identified with known co-morbid conditions. Specifically, 6 patients had diabetes mellitus (DM), 12 had hypertension (HTN), and 4 had both DM and HTN. Other recorded co-morbidities included atrial fibrillation in one patient, coronary artery disease (CAD) with DM and hypertension in five, hypothyroidism in five, chronic renal disease in three, and chronic liver disease in two. Among the paediatric category, one child exhibited disorders of sexual development (DSD), while another child had thalassemia major (Table 2). Statistical analysis revealed that 2 pediatric patients and 38 adult patients had known co-morbid conditions, and this difference was deemed statistically significant with a p-value of 0.03.

**Table 2:** Co-morbidities in patients with inguino-scrotal or labial swellings. DM: diabetes mellitus, HTN: hypertension, CAD: coronary artery disease, CKD: chronic kidney disease, DSD: disorder of sexual development.

Co-morbidities	Number of patients
DM	6
HTN	12
Diabetes and hypertension	4
CAD, along with diabetes and hypertension	5
CKD associated with hypertension	3
Chronic liver disease	2
Hypothyroidism	5
Atrial fibrillation	1
DSD	1
Thalassemia major	1

The final diagnosis for all 210 cases was determined based on the information collected during the clinical examination, as tabulated in Table 3.

**Table 3:** Diagnosis of patients with inguino-scrotal/labial swellings and detection on clinical parameters. ^a^n (%). N: total number of patients; Yes: the condition was diagnosed/present in the patient; No: the condition was not diagnosed; DD: differential diagnoses/aetiology.

Characteristics	N = 210^a^	Female, N = 16^a^	Male, N = 194^a^	Pediatric, N = 84^a^	Adult, N = 126^a^	Clinical diagnosis
Final diagnosis of swelling						
Inguinal abscess	2 (0.95%)	0 (0.00%)	2 (1.03%)	1 (1.19%)	1 (0.79%)	Yes
Appendicular lump	1 (0.48%)	0 (0.00%)	1 (0.52%)	0 (0.00%)	1 (0.79%)	Yes
Cellulitis	1 (0.48%)	0 (0.00%)	1 (0.52%)	1 (1.19%)	0 (0.00%)	Equivocal - inconclusive (DD: abscess)
Epididymal cyst	1 (0.48%)	0 (0.00%)	1 (0.52%)	0 (0.00%)	1 (0.79%)	Equivocal - inconclusive (DD: mass)
Epididymitis	1 (0.48%)	0 (0.00%)	1 (0.52%)	1 (1.19%)	0 (0.00%)	Equivocal - inconclusive (DD: torsion)
Epididymo-orchitis	1 (0.48%)	0 (0.00%)	1 (0.52%)	0 (0.00%)	1 (0.79%)	Equivocal - inconclusive (DD: torsion)
Fournier's gangrene	3 (1.43%)	0 (0.00%)	3 (1.55%)	0 (0.00%)	3 (2.38%)	Yes in 2; No in 1 - hernia associated.
Hernia	150 (71.43%)	14 (87.50%)	136 (70.10%)	45 (53.57%)	105 (83.33%)	Yes - 95% cases
Hydrocele	29 (13.81%)	0 (0.00%)	29 (14.95%)	19 (22.62%)	10 (7.94%)	Yes
Inguinal lymphadenopathy	2 (0.95%)	2 (12.5%)	0 (0.00%)	1 (1.19%)	1 (0.79%)	No
Scrotal oedema with hydrocele	1 (0.48%)	0 (0.00%)	1 (0.52%)	0 (0.00%)	1 (0.79%)	No
Testicular carcinoma	2 (0.95%)	0 (0.00%)	2 (1.03%)	1 (1.19%)	1 (0.79%)	No
Testicular torsion	7 (3.33%)	0 (0.00%)	7 (3.61%)	7 (8.33%)	0 (0.00%)	Equivocal - inconclusive due to Infective aetiology
Undescended testes	9(4.28%)	0 (0.00%)	9 (4.63%)	9 (10.71%)	0 (0.00%)	Equivocal

**Figure 1:**
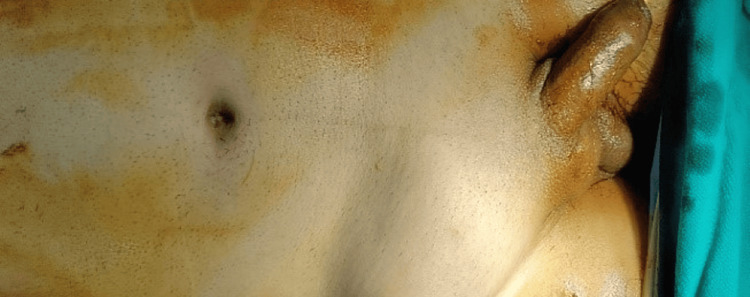
Right Amyand's hernia with caecum, appendix, the undescended testis and omentum as its content. Age: 32 years, sex: male.

We encountered several intriguing cases where radiology's contribution played a crucial role in achieving a pre-operative diagnosis. A notable example involved an emergency scenario with an irreducible hernia, revealing the presence of an undescended testis, along with the appendix, caecum, and omentum (Amyand's hernia). Sonography played a pivotal role in providing valuable insights, as depicted in Figure 1, leading to the determination of this complex condition.

**Figure 2:**
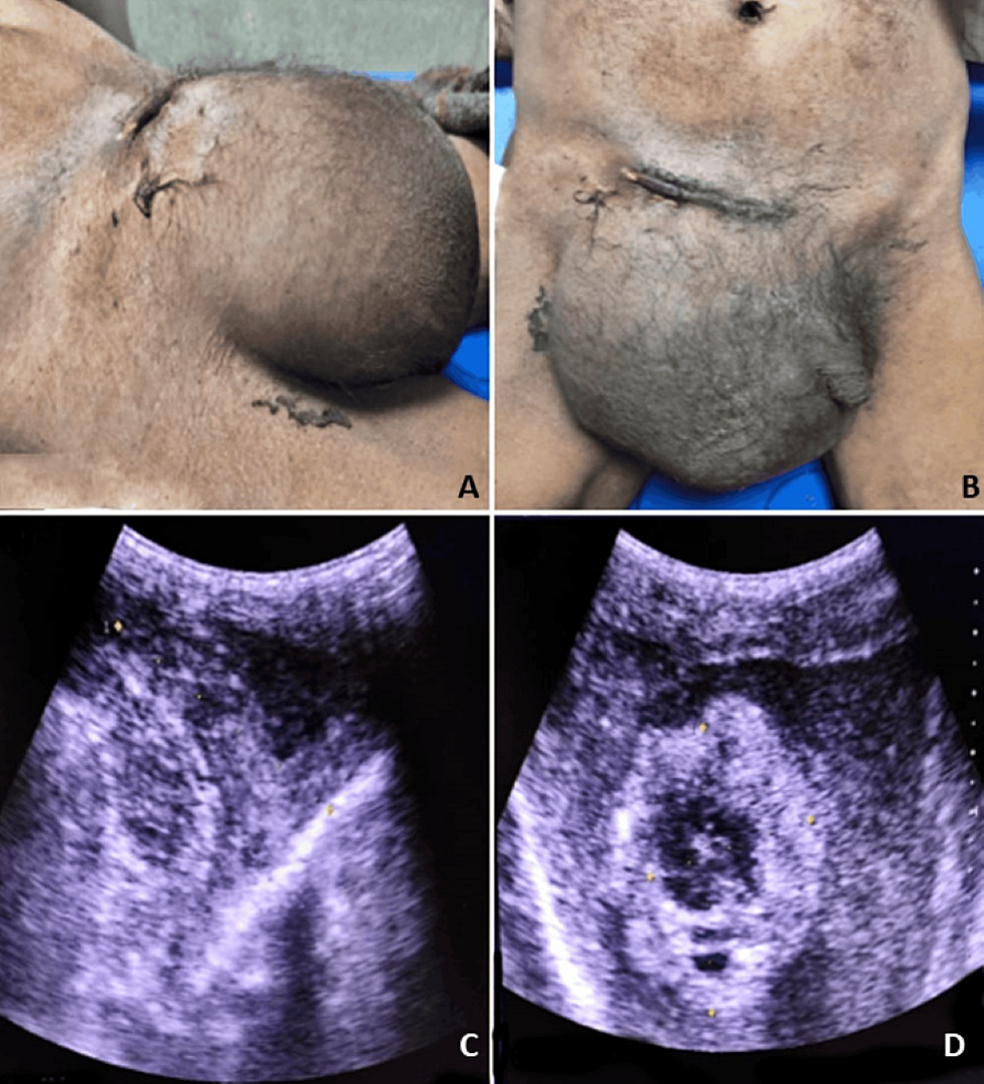
Giant right recurrent inguinal hernia immediately post-operation along with ultrasound images done on the same patient. (A, B) Gross appearance of recurrent inguinal hernia immediately post-operation; (C, D) ultrasound images of recurrent inguinal hernia immediately post-operation. Age: 42 years, sex: male.

**Figure 3:**
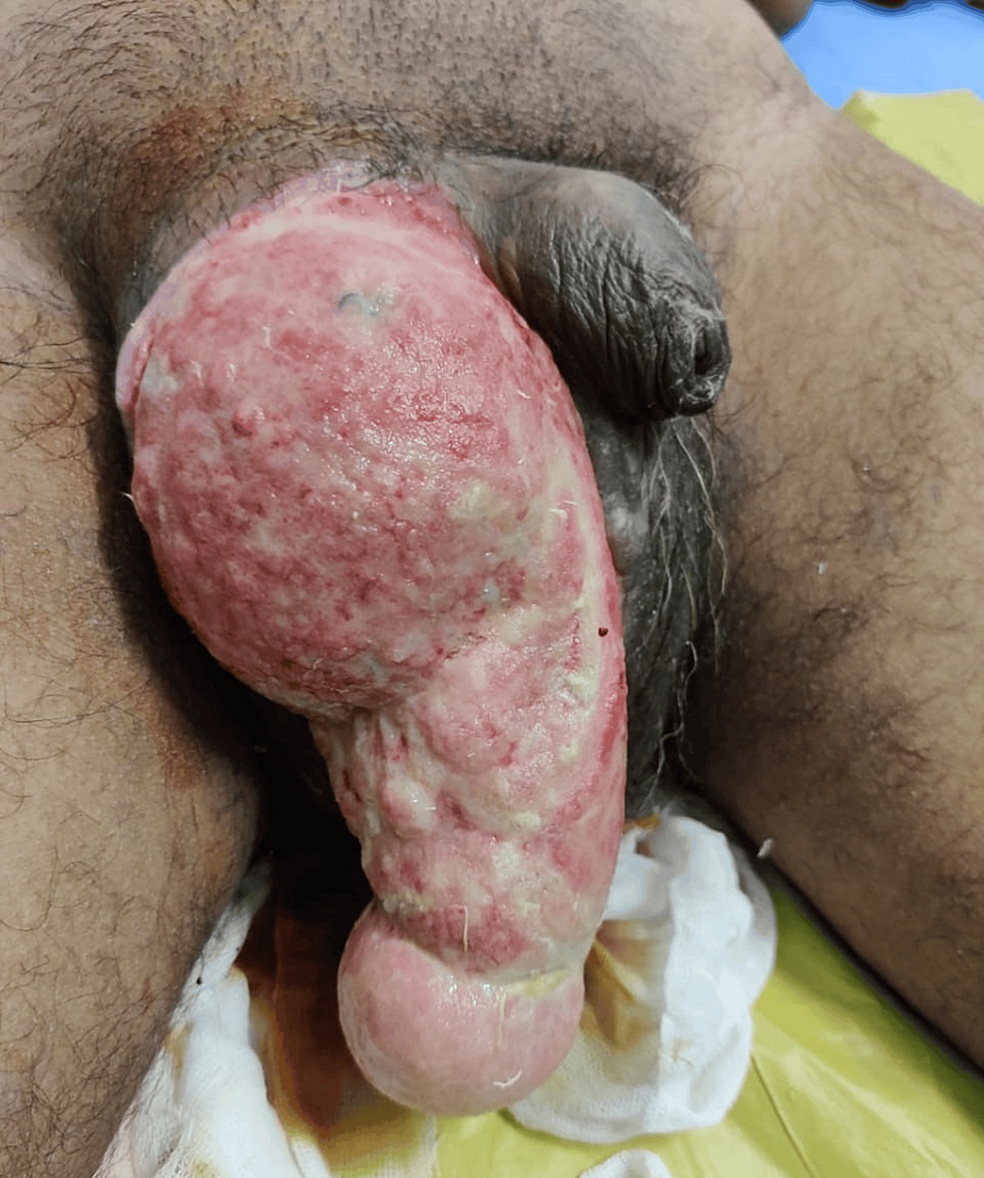
Inguinal hernia along with Fournier's gangrene. Age: 33 years, sex: male.

**Figure 4:**
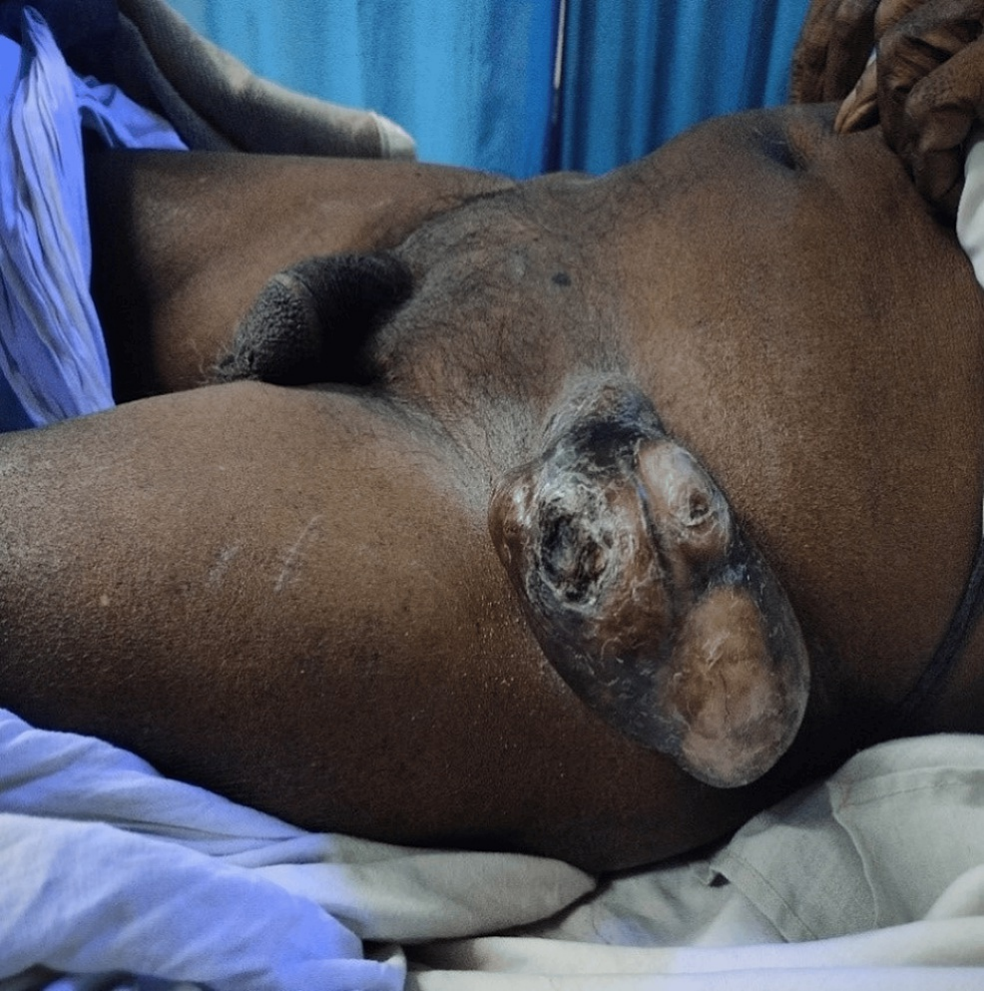
Scrofuloderma of inguinal region. Age: 36 years, sex: male.

**Figure 5:**
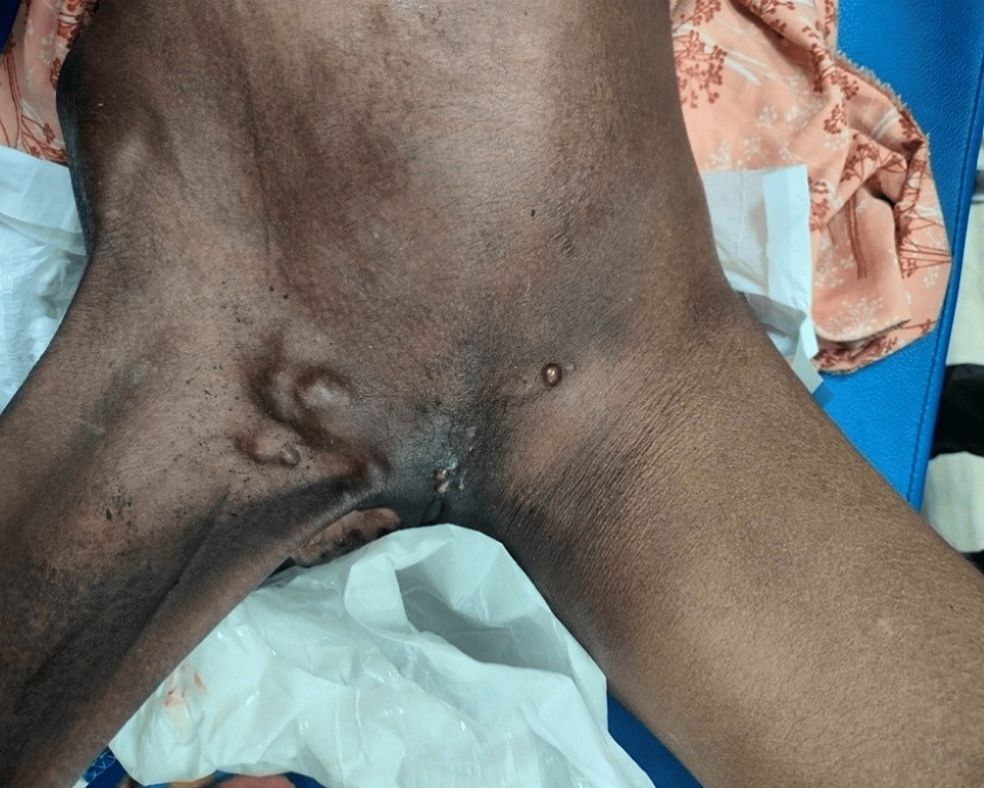
Inguinal lymph nodal secondaries from carcinoma of the anal canal. Age: 38 years, sex: male.

In Figure 2, the second case involved a patient who had previously undergone surgery for an inguinal hernia outside our institution. Upon arrival at our facility, the patient displayed signs of shock and presented with an irreducible, substantial swelling in the right inguinoscrotal area. The challenge was ascertaining whether it was a recurrence, an infected collection, or a significant hematoma contributing to the patient's condition. Sonography played a crucial role in confirming it as a recurrence. In situations where ambiguity persists, additional imaging modalities such as contrast-enhanced computed tomography (CECT) or MRI might be deemed necessary. Radiological evaluation proves essential in effectively deciphering such postoperative complications. Figure 3 illustrates an unusual case of Fournier’s gangrene (clinically diagnosed) with a co-existent inguinal hernia. In Figure 4, there was another case of a fungating lesion over the groin, with the biopsy revealing it to be scrofuloderma (on histopathology examination). One of the common findings in various perineal malignancies is inguinal lymph node secondaries (histo-pathological diagnosis), depicted in Figure 5.

Role of radiology in the study

Out of the 210 participants, radiological investigations played a crucial role in diagnosing conditions in 40 individuals (19.04%). Among the participants, 4 out of 16 female patients (25%) and 36 out of 194 male patients (18.56%) required radiological investigations for their diagnoses. Moreover, these investigations significantly contributed to the diagnosis in 12 patients (5.71%). Therefore, radiological investigations were not only essential for the diagnosis of 40 patients but also provided crucial additional information for the diagnosis of another 12 patients. In total, 52 patients out of 210 (24.76%) demonstrated a positive contribution from radiological aid. This study has uncovered various new entities in the inguinoscrotal/labial region, highlighting the limitations of relying solely on clinical parameters for diagnosis. These entities include malignancies, lymph nodal masses in the groin, vascular issues, inflammatory conditions, and a few congenital lesions, all of which might have been overlooked without the assistance of radiological evaluations. The role of radiology has been tabulated in Table 4, showing its diagnostic significance in terms of its contribution to the confirmatory diagnoses of the condition.

**Table 4:** Role of radiology in inguinoscrotal/labial swellings in the study. N: number of patients; Diagnostic: Radiology played the role of confirmatory diagnoses; Contributory: radiology just had a contributory role in the diagnoses (not confirmatory); Diagnostic significance: role of radiology in diagnoses (confirmatory/contributory).

Characteristic	N = 210	Female, N = 16	Male, N = 194	Pediatric, N = 84	Adult, N = 126
Diagnostic	40 (19.04%)	4 (25%)	36 (18.55%)	4 (4.76%)	36 (28.57%)
Contributory	12 (5.71%)	0 (0.00%)	12 (6.18%)	9 (10.71%)	3 (2.38%)
Diagnostic significance	Total = 52 (24.76%)	Total = 4 (25%)	Total = 48 (24.74%)	Total = 12 (14.28%)	Total = 39 (30.95%)

## Discussion

Inguino-scrotal/labial swellings are common in surgical OPD. The causes are diverse, with clinical examination being the primary evaluation method. Given the complexity of cases reaching tertiary centres, such as intricate swellings, emergencies, and malignancies, it is advisable to supplement clinical assessment with radiological modalities to evaluate inguino-scrotal/labial swellings comprehensively. This observational study aims to elucidate the role of radiology in assessing these swellings in a tertiary care hospital. The study predominantly focuses on male participants with inguino-scrotal/labial swellings, aligning with the gender distribution observed in similar studies. The presentation of inguinal swellings varies significantly, necessitating careful evaluation for diagnostic purposes. Some cases lack distinct characteristics for a definitive diagnosis, making radiological guidance essential. This research reveals a higher occurrence of female inguinal hernias than previously reported data [1,6]. The larger sample size in this study enhances its representativeness. Notably, approximately 71% of the patients exhibited an indirect type of hernia, consistent with findings in other comparable studies. In a prior study within the same geographical region, Agarwal noted a 60% presentation of indirect hernias, while Rao et al. reported 83.61% [7,8].

In this study, the predominant presentation among patients with inguino-scrotal/labial swellings was inguinal hernia, accounting for 71.4% of the total study population (150 out of 210 patients). Ultrasound examination is a highly sensitive modality for assessing inguinal hernias, especially when clinical examination results are inconclusive. Hernias, characterized by tissue with variable echogenicity, protrude from the inguinal ligament. The location of a direct inguinal hernia is medial to the inferior epigastric artery, while an indirect hernia appears lateral to the artery. Additionally, femoral hernias can be readily identified. Ultrasound aids in investigating potential aetiologies such as intra-abdominal masses or an enlarged prostate. Doppler assessment can detect strangulation in cases of incarcerated hernias. A study by Verma et al. in a tertiary centre focused on paediatric patients yielded similar findings, with inguinal hernia being the predominant diagnosis in most cases (72.7%) [9-12].

Radiological evaluation is crucial for cases of hydrocele, particularly when clinical assessment of the testis is challenging. Pyoceles may exhibit internal echoes, and ultrasound can reveal locations or septations even in chronic hydroceles. Abdomino-scrotal hydroceles might require a CT scan to assess extension into the abdomen and identify any communication with bowel or solid organs [13]. Except for large inguinal canal lipomas associated with inguinal hernias, ultrasound is generally adequate for diagnosis based on echogenicity and the absence of peritoneal connection. Lipomas typically present as heterogeneous, hypoechoic masses with minimal Doppler flow [14]. Malignant lesions, on the other hand, appear hyperechoic with necrotic components, while benign lesions exhibit well-defined boundaries. Coronal segments of CT scans can clearly depict the posterolateral emergence of these lipomas alongside the cord [15]. Spermatoceles, typically unilocular and clinically identifiable, may pose a diagnostic challenge with multilocular cysts, where ultrasound proves beneficial. Ultrasound is also employed in evaluating undescended testes, identified by their small, hypoechoic nature, and MRI may be utilized for locating intra-abdominal or retroperitoneal positions.

Ultrasound proves to be a valuable diagnostic tool for identifying subclinical varicosities that may not be apparent during a clinical examination. It also facilitates the assessment of testicular volume, considering the debated association between varicoceles and impaired spermatogenesis, leading to oligozoospermia and infertility [16]. Furthermore, ultrasound assists in evaluating the kidneys to detect any renal tumours contributing to varicocele development. Doppler assessment helps grade varicoceles based on reflux varicosities and testicular hypotrophy. In differentiating epididymal orchitis from testicular torsion, a challenging and similar presentation, ultrasound plays a crucial role.

For patients with a history of trauma, colour flow Doppler aids in distinguishing between a vascular tumour and an intratesticular hematoma, which may coexist in some cases. The preferred method for assessing metastases is CECT of the chest, abdomen, and pelvis. Wani et al. [16] conducted a study on inguinoscrotal swellings in children, reporting hydrocele as the predominant diagnosis in 31 patients, followed by an inguinal hernia in 14 cases. Vinoth et al. [17] reported a case of unusual inguino-labial swellings in an adult female, highlighting the significance of radiological evaluation in atypical inguino-scrotal/labial swellings. Kojima et al. [18] demonstrated the diagnostic accuracy of ultrasonography findings in inguinal hernia surgeries. In the current study, a case of sliding Amyand hernia was observed, wherein the caecum formed one of the sac's walls, and the appendix, omentum, and undescended testis comprised its contents. In a study on clinicopathological and radiographic evaluation of scrotal swellings in Meerut conducted by Chauhan et al., three cases (6%) of epididymal cysts were identified among 50 patients. However, none were associated with testicular microlithiasis [19,20]. A diagnostic challenge involving femoral hernia, inguinal hernia with irreducibility, lipoma, lymphangioma, leiomyoma, sarcoma, and occasionally round ligament varicosities, cysts, abscesses, and lymph nodes may necessitate the use of imaging modalities [21]. Ultrasound is the most commonly used modality and is proficient at detecting the presence of bowel loops, ovaries, ectopic pregnancy, or endometrial tissue in the inguinal canal. Further investigations, such as CT scans or MRIs, may be warranted if ultrasound falls short in elucidating findings. In children, MRI is preferred due to its lack of radiation exposure, especially when a solid lesion is strongly suspected in the differential diagnosis [22].

In this study, the role of radiology proved pivotal in diagnosing 20 cases solely based on radiological findings while also playing a confirmatory role in 12 cases where imaging was instrumental in validating the diagnosis. The statistical significance of radiology in certain diagnoses contributed to increased diagnostic accuracy. Kojima et al. reported a diagnostic accuracy of 92.7% for imaging of inguinal hernias, reinforcing the reliability of radiological assessments. Notably, two cases in this study were diagnosed and confirmed separately by radiological findings and operated on via open herniotomy. Additionally, three cases were exclusively diagnosed by radiology and subsequently treated with open hernioplasty. The study identified seven cases (3.33%) of testicular torsion, all within the paediatric age group, highlighting the importance of radiological evaluation. Sim and Park conducted a study with a similar lack of cases of testicular torsion in children [23]. Furthermore, two cases (0.95%) of lipoma of the cord coexisting with inguinal hernia were observed in this study. A reported case of a large lipoma of the cord mimicking inguinoscrotal hernia in a 62-year-old male underscores the significance of sonography evaluation and the complementary role of MRI imaging in such cases [24]. Out of 210 patients with inguino-scrotal/labial swellings, 9 cases (4.28%) of undescended testicles were identified, with only one case also exhibiting testicular torsion during sonography analysis for congenital foetal defect (CFD) [25, 26]. Three cases (1.43%) of Fournier's gangrene were identified in the course of this study, with one case also having an inguinal hernia. While Fournier's gangrene is primarily a clinical diagnosis, the presence of uncommonly associated manifestations necessitates radiological assessment to avoid potential complications. Other studies, such as Kundan et al., have reported cases of Fournier's gangrene in conjunction with a sliding inguinal hernia [27]. Neglected Richter's inguinal hernia leading to Fournier's gangrene has also been reported in the literature [28].

Two cases (0.95%) of femoral hernias were identified in this study, both in adult males, even though female patients are more commonly associated with this condition. Sonography examinations play a crucial role in visualizing hernias, aiding in the assessment of common complications like incarcerated hernia and obstruction or strangulation. The study emphasizes the importance of pre-operative diagnosis for better management planning and patient counselling. A case of left epididymal cyst with bilateral testicular microlithiasis, initially presenting as left testicular enlargement and persistent bilateral testicular pain, is also highlighted in this study.

Limitations

A notable limitation of the current study is the expected lopsided gender distribution, which introduces a potential bias that could impact the generalizability of the findings. Additionally, the study's focus on a specific region in India (Central India with few referrals from surrounding states) limits the extrapolation of results to a national context. Future investigations should adopt a multicenter approach encompassing diverse regions across the country to obtain a more holistic perspective. Another limitation lies in the predominantly adult participant demographic, overlooking potential variations in inguino-scrotal/labial swellings among the paediatric age group. Expanding the study's scope to include a more diverse age range would enhance the overall understanding of the condition. Furthermore, considering alternative statistical methods to evaluate a composite score derived from various indicators could contribute to a more nuanced analysis, bolstering the robustness and applicability of the study's outcomes.

## Conclusions

In conclusion, our findings underscore the crucial role of radiological assessment in patients presenting with inguino-scrotal/labial swelling. It should be deemed an essential criterion for managing selected cases, particularly when clinical findings are inconclusive or fail to provide a definitive diagnosis. This is significant in tertiary care centres where advanced radiological facilities are readily accessible. Moreover, advocating for extending radiological assessments to other levels of healthcare could significantly enhance diagnostic accuracy, contribute to the prevention of complications, and ultimately lead to improved patient management. Emphasizing the integration of radiological tools in the evaluation of inguino-scrotal/labial swellings can optimize healthcare outcomes and enhance the overall quality of patient care.
